# The Efficacy and Safety of Combining Cross‐Linked Hyaluronic Acid Filler VYC‐12L and Energy‐Based Devices for Facial Skin Quality Improvement in Asians

**DOI:** 10.1111/jocd.70485

**Published:** 2025-10-09

**Authors:** Mikako Oku, Kentaro Oku

**Affiliations:** ^1^ Hills Grace Clinic Yokohama Japan

**Keywords:** collagen synthesis, combination therapy, cross‐linked hyaluronic acid (CL‐HA) filler, energy‐based devices (EBDs), facial aging, skin quality improvement

## Abstract

**Background:**

In recent years, treatments using energy‐based devices (EBDs) aimed at skin quality improvement have become widely adopted. However, it has been pointed out that treatments using EBDs alone show limited durability of effects and variability in patient‐perceived improvements. Therefore, we evaluated skin quality improvement resulting from a combination therapy involving cross‐linked hyaluronic acid (CL‐HA) filler and EBD treatments tailored to patients' chief concerns.

**Objective:**

To assess the efficacy and safety of combined CL‐HA filler and EBD therapies in treating facial skin quality improvement among Asian subjects.

**Methods:**

In this prospective, evaluator‐blinded study, 18 participants aged 32–59 years with decreased skin elasticity, fine wrinkles, uneven texture, pore enlargement, pigmentation, and sagging were enrolled. CL‐HA filler (VYC‐12L) was injected intradermally into the entire face. One month later, according to each patient's primary concerns and symptoms, EBD treatment (either high intensity, high frequency parallel ultrasound beam [PUB], picosecond alexandrite laser [PSAL], and sequential monopolar bipolar pulsed radiofrequency [SMBPRF]) was administered to the entire face. Evaluations were conducted at three time points: baseline (T0), 4 weeks post‐CL‐HA injection (T1), and 8 weeks post‐EBD treatment (T2). Subjective assessment was performed using the FACE‐Q | Aesthetics—Satisfaction with Skin scale. Objective assessment was independently conducted by a dermatologist utilizing three‐dimensional image analysis and skin diagnostic imaging with the Global Aesthetic Improvement Scale (GAIS).

**Results:**

All participants completed the study. Pairwise comparisons of the FACE‐Q skin satisfaction scores demonstrated statistically significant improvements at all time points: from T0 to T1 (*p* < 0.05), from T0 to T2 (*p* < 0.001), and from T1 to T2 (*p* < 0.001). Objective assessment with the GAIS showed improvement in all cases: very much improved (2 cases), much improved (9 cases), improved (7 cases), no change (0 cases), and worsened (0 cases). Regarding safety, no severe adverse events were reported, and only minor transient post‐treatment reactions were observed.

**Conclusions:**

The combination of CL‐HA filler and EBD represents a safe and effective therapeutic approach for enhancing facial skin quality in Asian subjects.

## 1 Introduction

In recent years, the demand for non‐invasive skin quality improvement treatments in aesthetic dermatology has been rapidly increasing. In particular, treatments utilizing energy‐based devices (EBDs), such as ultrasound, radiofrequency, and laser, have become widely employed. These therapies aim to thermally stimulate fibroblasts within the dermis, promoting the production of extracellular matrix components such as collagen, elastin, and mucin. However, treatments using EBDs alone have been associated with a limited duration of efficacy and notable inter‐individual variability in perceived improvements. One reason for these limitations is the insufficient functional recovery of fibroblasts, which are chronically damaged due to ultraviolet exposure and aging. Consequently, EBD treatment alone may fail to adequately stimulate extracellular matrix production [1, 2, 3].

Ultraviolet exposure, particularly ultraviolet A (UVA), is deeply involved in skin photoaging. UVA exposure increases reactive oxygen species (ROS) production in dermal fibroblasts, activating matrix metalloproteinases (MMPs) that accelerate collagen fiber degradation. Furthermore, chronic UVA exposure induces fibroblast senescence, significantly reducing their physiological functions and collagen synthesis capabilities. Moreover, senescent fibroblasts can promote the aging of neighboring cells and tissues via senescence‐associated secretory phenotype (SASP), exacerbating overall skin aging [4].

To address chronic fibroblast damage and functional decline, intradermal injection of CL‐HA fillers has gained attention as a novel therapeutic option. It has been reported that CL‐HA fillers induce mechanical stretching of dermal fibroblasts upon intradermal injection, activating the TGF‐β signaling pathway [5, 6].

Additionally, injection of CL‐HA fillers has been shown to enhance moisture retention in the epidermis surrounding the injection site, which may improve tissue tolerance to thermal stimuli induced by EBD treatments [7].

The purpose of this study is to evaluate the clinical efficacy and safety of combination therapy using CL‐HA filler and EBDs.

## 2 Materials and Methods

This prospective, evaluator‐blinded study was performed to subjectively and objectively evaluate skin quality improvement resulting from combined treatment using CL‐HA filler and EBDs in accordance with the Declaration of Helsinki.

The subjects included 18 female patients aged 32 to 59 years who sought aesthetic treatment for complaints of loss of firmness, decreased skin elasticity, fine wrinkles, uneven skin texture, pigmentation, sagging, loss of firmness, and enlarged pores. Exclusion criteria included active infection at the treatment sites, severe dermatological disease (premalignant cutaneous lesions including actinic keratosis [AK] and malignant cutaneous lesions), a history of keloid formation, autoimmune disease, pregnancy, and breastfeeding. Subjects participated in the study from November 2023 to February 2025. Signed informed consent was obtained from each participant before enrollment.

The CL‐HA filler used in this study was VYC‐12L (Juvederm Vista Volite XC; Allergan Aesthetics, an AbbVie company, Irvine, CA, USA), which contains cross‐linked hyaluronic acid at a concentration of 12 mg/mL and lidocaine 0.3%. VYC‐12L was uniformly injected intradermally into the entire face (total volume 3 cc: 1 cc each in bilateral cheeks, 1 cc in the forehead area), but not into the submental area. After injection, a waiting period of approximately 1 month was provided to facilitate tissue integration of the CL‐HA filler.

After this waiting period, participants underwent treatment using one of the following three types of EBDs, selected based on their primary skin concerns:

(1) For participants primarily concerned with fine wrinkles, uneven skin texture, and enlarged pores:

High‐intensity, high‐frequency parallel ultrasound beam; PUB (Sofwave; Sofwave Medical Ltd., Yokneam, Israel) was applied to the entire face using an energy setting of 3.0–3.2 J/shot with the Thermal‐Thread Technique [8].

(2) For participants primarily concerned with loss of firmness and sagging:

Sequential monopolar and bipolar pulsed radiofrequency; SMBPRF (Density; Jeisys Medical Inc., Seoul, Korea) was applied to the entire face using the High‐F tip, which simultaneously delivers monopolar and bipolar radiofrequency energy in a single shot. The energy level was set at a range (1.5–3.0) at which participants perceived thermal sensation without experiencing pain.

(3) For participants primarily concerned with superficial benign pigmented lesions:

Picosecond alexandrite laser; PSAL (PICOSUREpro; Cynosure LLC., Westford, MA, USA) was used. Individual pigmented lesions were treated using the flat beam (ZOOM) at fluences of 2.2–2.5 J/cm^2^, followed by fractional irradiation to the entire face using a diffractive lens array (FOCUS). This fractional treatment was performed at approximately 9 J/cm^2^, a threshold fluence sufficient to induce laser‐induced optical breakdown (LIOB) [9].

Subjective evaluation was quantified using FACE‐Q | Aesthetics—Satisfaction with Skin (Memorial Sloan Kettering Cancer Center, NY, USA), measuring improvements in skin texture, elasticity, hydration, glowing, tones, pores, color, and fine wrinkles (score range: 0–100). These assessments were conducted at three time points during the study: at baseline (T0), 4 weeks after the CLHA filler injection (T1), and 8 weeks after the EBD treatment (T2).

Objective evaluations were conducted using three‐dimensional imaging (VECTRA H2; Canfield Scientific, Parsippany, NJ, USA) and high‐resolution skin diagnostic imaging (Re‐Beau 2; JMEC Co. Ltd., Tokyo, Japan), capturing images at T0 and T2. Images were evaluated by an independent dermatologist using the Global Aesthetic Improvement Scale (GAIS) rated on a 5‐point scale: very much improved, much improved, improved, no change, or worsened.

Statistical analysis was performed using Python (version 3.8; Python Software Foundation, Wilmington, DE, USA). Pairwise changes in FACEQ scores (T0–T1, T0–T2, and T1–T2) were analyzed using two‐sided paired t‐tests via the SciPy library (version 1.10.1; SciPy Developers). A *p*‐value of < 0.05 was considered statistically significant.

For safety assessment, adverse reactions (e.g., edema, bruising, pain) occurring during or after treatment with the CL‐HA filler and EBDs were recorded.

## 3 Results

A total of 18 patients, all females, participated in this study, with a mean age of 47.9 ± 8.2 years (range: 32–59 years). The distribution of Fitzpatrick skin types was as follows: type II in 5 cases, type III in 12 cases, and type IV in 1 case. The energy‐based devices used were PUB in 8 cases, SMBPRF in 5 cases, and PSAL in 5 cases (Table 1).

**TABLE 1:** Patient characteristics and the results of FACE‐Q score and GAIS.

Case number	Age	FST	Chief complaint	EBDs	FACE‐Q			GAIS (T2)
					T0	T1	T2	
1	32	2	Texture irregularity	Sofwave	21	72	79	Much improved
2	59	2	Wrinkle, texture irregularity	Sofwave	29	49	74	Much improved
3	35	3	Pore, texture irregularity	Sofwave	34	51	74	Much improved
4	36	2	Pigmentations, texture irregularity	PICOSUREpro	18	81	88	Very much improved
5	40	3	Pigmentations, texture irregularity	PICOSUREpro	39	63	84	Much improved
6	40	3	Sagging, loss of firmness	DENSITY	47	55	69	Much improved
7	57	3	Wrinkle, texture irregularity	Sofwave	34	51	72	Improved
8	53	2	Wrinkle, texture irregularity	Sofwave	34	55	74	Very much improved
9	48	3	Pigmentations, pore	PICOSUREpro	43	45	63	Improved
10	59	3	Wrinkle, texture irregularity	Sofwave	14	22	39	Improved
11	52	3	Pigmentations, texture irregularity	PICOSUREpro	36	49	57	Much improved
12	44	2	Wrinkle, texture irregularity	Sofwave	43	50	51	Much improved
13	47	4	Sagging, texture irregularity	DENSITY	32	38	43	Much improved
14	49	3	Texture irregularity	Sofwave	29	36	39	Improved
15	48	3	Sagging, loss of firmness	DENSITY	47	50	57	Improved
16	53	3	Sagging, loss of firmness	DENSITY	39	44	55	Improved
17	55	3	Pigmentations, texture irregularity	PICOSUREpro	45	47	51	Much improved
18	55	3	Sagging, texture irregularity	DENSITY	36	51	57	Improved

**FIGURE 1:**
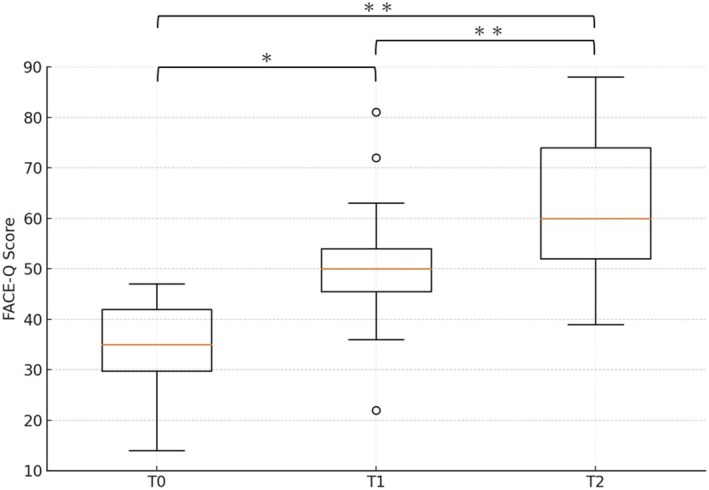
Box‐and‐whisker plots of the FACE‐Q | Aesthetics—Satisfaction with Skin score at baseline (T0), 4 weeks after the CL‐HA filler injection (T1), and 8 weeks after the EBD treatment (T2). Brackets indicate pairwise comparisons assessed with two‐sided paired *t*‐tests. Asterisks indicate statistical significance: *p < 0.05; **p < 0.001.

Pairwise changes in the FACE‐Q skin satisfaction score (T0–T1, T0–T2, and T1–T2) showing significant increases from T0 to T1 (Δ = 16.06, *t*(17) = 4.13, *p* < 0.05, 95% CI [7.86, 24.26]), T0 to T2 (Δ = 28.11, *t*(17) = 6.46, *p* < 0.001, 95% CI [18.92, 37.30]), and T1 to T2 (Δ = 12.06, *t*(17) = 6.64, *p* < 0.001, 95% CI [8.23, 15.89]) (Figure 1).

**FIGURE 2:**
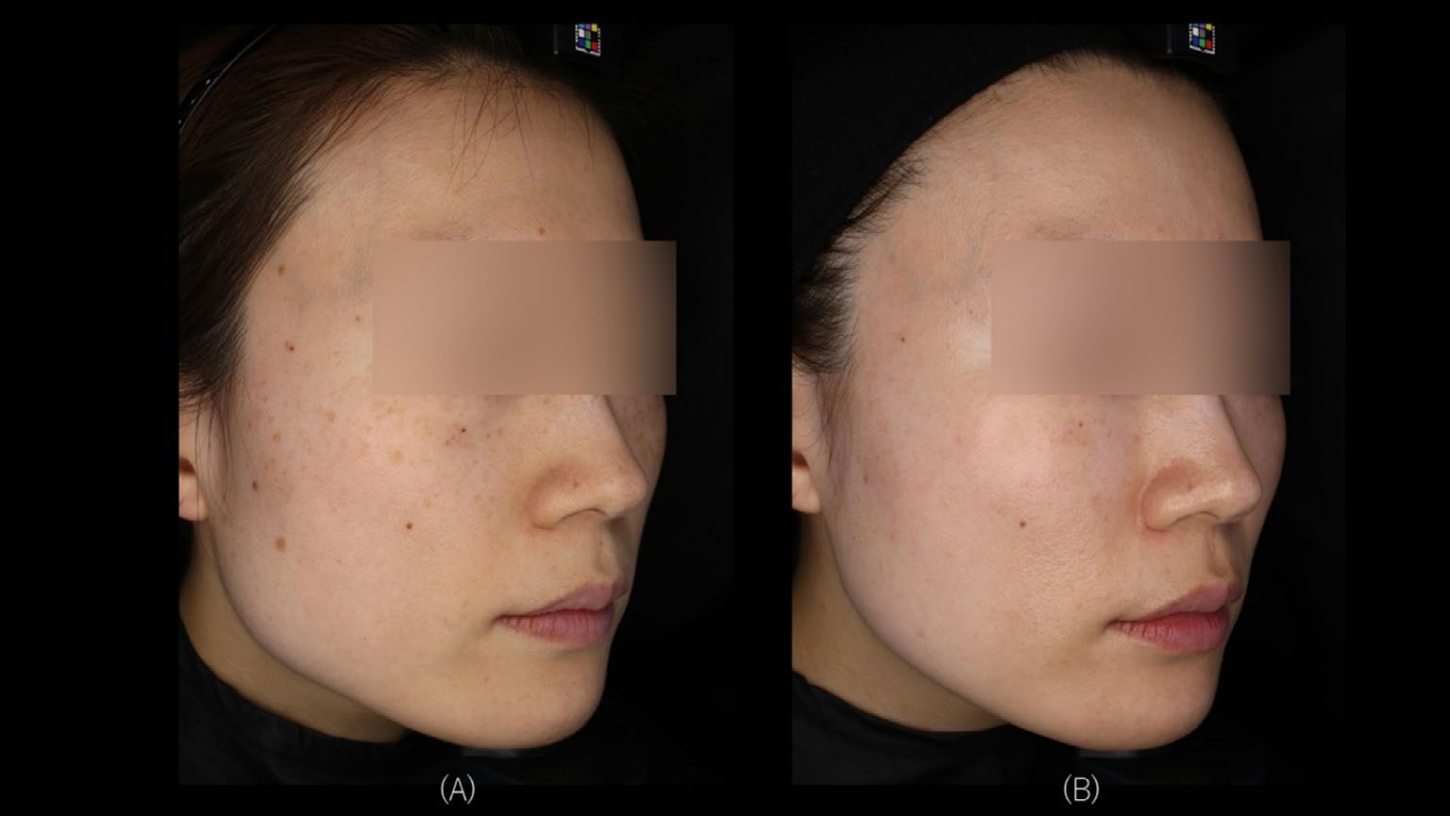
A 36‐year‐old female with Fitzpatrick Skin Type II. Treated with VYC‐12L and Picosecond Alexandrite laser. (A) Right side view at T0. (B) Right side view at T2.

**FIGURE 3:**
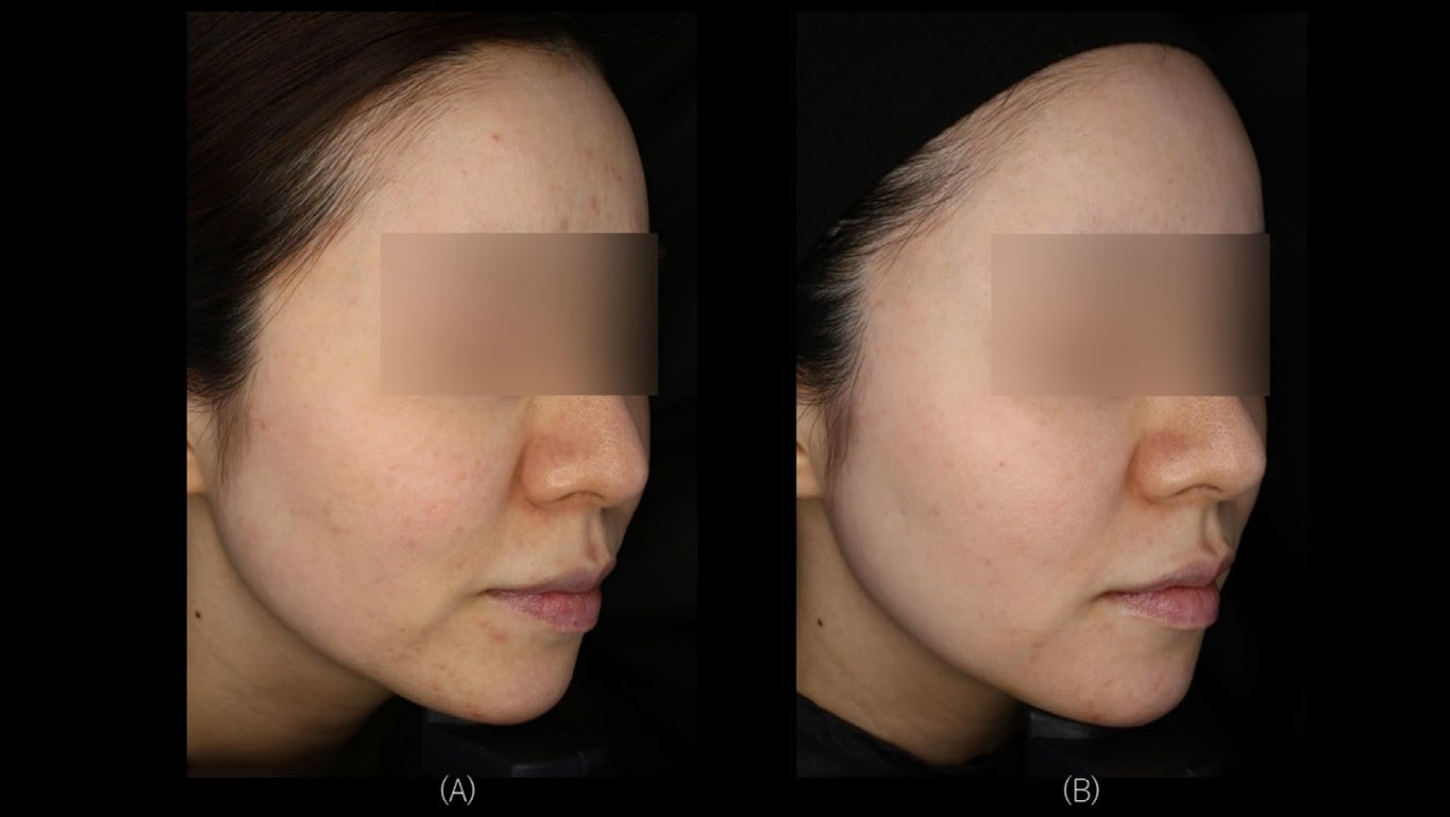
A 40‐year‐old female with Fitzpatrick Skin Type III. Treated with VYC‐12L and Sequential Monopolar and Bipolar Pulsed Radiofrequency. (A) Right side view at T0. (B) Right side view at T2.

**FIGURE 4:**
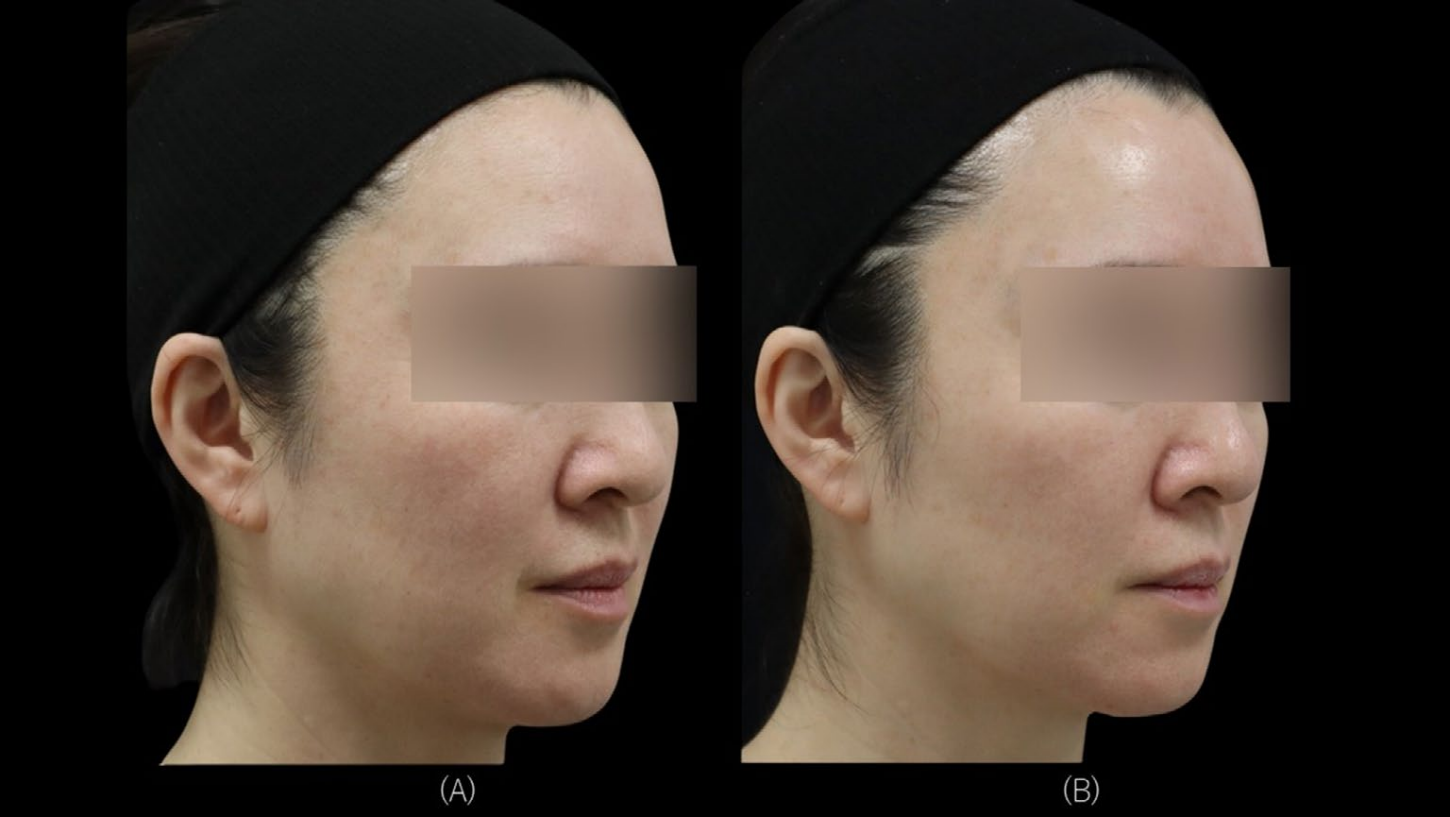
A 53‐year‐old female with Fitzpatrick Skin Type II. Treated with VYC‐12L and high intensity, high frequency parallel ultrasound beam. (A) Right side view at T0. (B) Right side view at T2.

Objective evaluation based on Global Aesthetic Improvement Scale (GAIS), assessed by an independent dermatologist using images obtained from three‐dimensional imaging (VECTRA H2; Canfield Scientific, Parsippany, NJ, USA) and high‐resolution skin diagnostic imaging (Re‐Beau 2; JMEC Co. Ltd., Tokyo, Japan), demonstrated improvements in all cases. Representative cases for each device are presented (Figures 2, 3, 4). Specifically, two patients were assessed as “very much improved,” nine patients as “much improved,” and seven patients as “improved.” None of the cases were assessed as “no change” or “worsened.”

Regarding safety, transient mild adverse reactions, including edema and bruising, were observed in a few cases immediately post‐treatment. However, these reactions resolved completely within 1 week. No cases experienced persistent pain, severe adverse events, or long‐lasting adverse effects.

## 4 Discussion

In the present study, it was demonstrated that combination treatment using the CL‐HA filler and EBDs resulted in significant improvements in skin texture, firmness, elasticity, fine wrinkles, pore dilatation, pigmentations, and sagging, accompanied by high patient satisfaction. These outcomes are likely attributable to the synergistic effect of the distinct mechanisms of action associated with each treatment modality.

EBDs primarily deliver thermal energy into deeper layers of the skin, stimulating collagen production by activating fibroblasts within the dermis via the Non‐SMAD pathway. However, conventional monotherapy using EBDs alone may be insufficient for fully restoring the functionality of fibroblasts that have been chronically damaged due to ultraviolet A (UVA) exposure and senescence. Consequently, the durability of clinical improvement and the extent of perceived patient benefit are thought to be limited when using EBD treatment alone.

Conversely, the CL‐HA filler utilized in this study, VYC‐12L, provides mechanical stretching stimuli to fibroblasts when injected into the dermis. This mechanical stimulation has been reported to enhance secretion of transforming growth factor‐beta 1 (TGF‐β1) and TGF‐β3, subsequently activating TGF‐β receptors (TβRII and TβRI) on the cell membrane and inducing phosphorylation of SMAD2 and SMAD3. Phosphorylated SMAD2/3 then forms a complex with SMAD4 and translocates to the nucleus, promoting transcription of connective tissue growth factor (CTGF) and type I procollagen genes. Prior studies have indicated that activation of this TGF‐β signaling pathway, the SMAD pathway, results in increased collagen synthesis and remodeling of dermal architecture. These molecular‐level mechanisms align well with the clinical skin improvements observed in our study. It is conceivable that the activation of the TGF‐β pathway through both the SMAD and Non‐SMAD pathways may have contributed to the progressive improvement in FACE‐Q scores observed over time. From the perspective of skin quality improvement, it may be considered that among the EBDs used in this study, the high‐intensity, high‐frequency parallel ultrasound beam (PUB) represents the most suitable combination with the CL‐HA filler.

Previous ultrasound imaging studies have demonstrated that a CL‐HA filler (VYC‐25), which has structural similarity to VYC‐12L, exhibits favorable tissue affinity within 48 h after injection and achieves complete biointegration within 1 month. Based on these reports, it is plausible that the CL‐HA filler used in our study similarly exhibited favorable tissue affinity and biointegration, thereby supporting and enhancing the therapeutic effects of the EBDs. Moreover, the increase in dermal hydration provided by HA filler injection may have reduced tissue damage induced by thermal stimulation during EBD treatments.

Furthermore, the enhanced skin hydration resulting from VYC‐12L injection may have contributed to reduced transient adverse reactions (such as erythema and skin dryness) commonly associated with EBD treatments. These combined mechanisms likely reduced post‐treatment downtime and discomfort, factors frequently noted as limitations of EBD monotherapy, thereby increasing overall patient satisfaction. However, it could also negatively affect the efficacy of EBDs by altering their depth of penetration; therefore, it should not be overlooked that careful adjustment of treatment depth and thermal dose is required when applying EBD.

Despite the promising findings, this study has several limitations. Firstly, the number of subjects assigned to each EBD treatment group was relatively small. Secondly, all participants were female, with the majority classified as Fitzpatrick Skin Type III; thus, our findings may not be generalizable to more diverse populations. Additionally, due to the variety of energy sources and treatment modalities used, the delivered energy might not exclusively target the dermal layer, making it difficult to precisely quantify the energy effectively reaching the dermis.

Future studies should employ a single device type to more accurately assess the efficacy of combined therapy. Furthermore, longer‐term follow‐up periods are warranted to comprehensively evaluate sustained therapeutic efficacy and safety. Additional research is also needed to optimize the timing and dosing protocols of EBD procedures and CL‐HA filler injections, ultimately aiming to establish a standardized treatment protocol.

In conclusion, combined treatment with CL‐HA filler and EBDs demonstrated significant efficacy in improving skin quality, including skin elasticity, texture, firmness, pigmentations, pores, and fine wrinkles, resulting in high patient satisfaction among Asian subjects. This dual‐modality approach offers a promising less invasive option for facial rejuvenation, effectively targeting multiple components implicated in skin aging, including collagen regeneration. Further research involving larger, more diverse populations and longer follow‐up periods is warranted to confirm these findings and to establish standardized protocols for this combined therapeutic strategy as a treatment for facial skin quality improvement.

## Author Contributions

All authors contributed significantly to the study conception and design. Material preparation, data collection, and analysis were performed by Kentaro Oku. The first draft of the manuscript was written by Mikako Oku, and all authors reviewed, edited, and approved the final manuscript.

## Ethics Statement

This study was conducted in compliance with the principles outlined in the Declaration of Helsinki. All participants provided written informed consent prior to enrollment.

## Conflicts of Interest

The authors declare no conflicts of interest.

## Data Availability

The data that support the findings of this study are available from the corresponding author upon reasonable request.
